# A *C. elegans* genome-wide RNAi screen for altered levamisole sensitivity identifies genes required for muscle function

**DOI:** 10.1093/g3journal/jkab047

**Published:** 2021-03-13

**Authors:** Timothy Chaya, Shrey Patel, Erin M Smith, Andy Lam, Elaine N Miller, Michael Clupper, Kirsten Kervin, Jessica E Tanis

**Affiliations:** 1 Department of Physiology, Perelman School of Medicine, University of Pennsylvania, Philadelphia, PA 19104, USA; 2 Department of Biological Sciences, University of Delaware, Newark, DE 19716, USA

**Keywords:** *C. elegans*, levamisole, acetylcholine, GABA, endocytosis, *epn-1*, *gas-1*, ATP

## Abstract

At the neuromuscular junction (NMJ), postsynaptic ionotropic acetylcholine receptors (AChRs) transduce a chemical signal released from a cholinergic motor neuron into an electrical signal to induce muscle contraction. To identify regulators of postsynaptic function, we conducted a genome-wide RNAi screen for genes required for proper response to levamisole, a pharmacological agonist of ionotropic L-AChRs at the *Caenorhabditis elegans* NMJ. A total of 117 gene knockdowns were found to cause levamisole hypersensitivity, while 18 resulted in levamisole resistance. Our screen identified conserved genes important for muscle function including some that are mutated in congenital myasthenic syndrome, congenital muscular dystrophy, congenital myopathy, myotonic dystrophy, and mitochondrial myopathy. Of the genes found in the screen, we further investigated those predicted to play a role in endocytosis of cell surface receptors. Loss of the Epsin homolog *epn-1* caused levamisole hypersensitivity and had opposing effects on the levels of postsynaptic L-AChRs and GABA_A_ receptors, resulting in increased and decreased abundance, respectively. We also examined other genes that resulted in a levamisole-hypersensitive phenotype when knocked down including *gas-1*, which functions in Complex I of the mitochondrial electron transport chain. Consistent with altered ATP synthesis impacting levamisole response, treatment of wild-type animals with levamisole resulted in L-AChR–dependent depletion of ATP levels. These results suggest that the paralytic effects of levamisole ultimately lead to metabolic exhaustion.

## Introduction

Acetylcholine (ACh), released from motor neurons, activates postsynaptic ionotropic ACh receptors (AChRs), resulting in an electrical signal that leads to muscle contraction. Disruption of cholinergic signaling at the neuromuscular junction (NMJ) is the underlying cause of severe muscle weakness observed in individuals with congenital myasthenic syndromes (CMS) and the autoimmune syndrome Myasthenia gravis (Engel *et al.* 2015). Clinical features of some congenital myopathies and muscular dystrophies resemble CMS despite different genetic causes and muscle histopathology (Mahjneh *et al.* 2013; Rodríguez Cruz *et al.* 2014; Montagnese *et al.* 2017). This suggests that characterizing mechanisms, which regulate postsynaptic cholinergic signaling, could provide an insight into multiple neuromuscular disorders.

*C. elegans* body wall muscles are functionally comparable to vertebrate skeletal muscles and provide an excellent model for the study of neuromuscular transmission (Gieseler 2017). There are two classes of ionotropic AChRs at the *C. elegans* NMJ that have distinct pharmacological profiles. L-AChRs are heteromeric AChRs that are sensitive to the nematode-specific AChR agonist levamisole, while N-AChRs are homomeric AChRs that are activated by nicotine, but insensitive to levamisole (Richmond and Jorgensen 1999; Touroutine *et al.* 2005). Wild-type animals exposed to levamisole undergo time-dependent paralysis (Lewis *et al.* 1980). Altered sensitivity to levamisole-induced paralysis can be indicative of defects in postsynaptic cholinergic signaling. A forward genetic screen for mutants with strong levamisole resistance identified mutations in 12 different genes encoding subunits of the L-AChR as well as proteins required for L-AChR trafficking, clustering, and muscle contraction (Lewis *et al.* 1980; Gally *et al.* 2004; Eimer *et al.* 2007; Gendrel *et al.* 2009). Levamisole-resistant mutants have also been isolated in a forward genetic screen for animals that initially exhibit paralysis in response to levamisole, but subsequently adapt and regain motility (Rapti *et al.* 2011; Boulin *et al.* 2012; Richard *et al.* 2013; D’Alessandro *et al*. 2018). However, mutants hypersensitive to levamisole, as well as levamisole-resistant mutants which also have a lethal or sterile phenotype, could not be isolated in these genetic screens. For example, loss of function mutations in *fer-1,* the *C. elegans* homolog of Dysferlin which is mutated in Limb-Girdle Muscular Dystrophy 2B, result in weak levamisole resistance, but also sterility (Achanzar and Ward 1997; Krajacic *et al.* 2013). Since *fer-1* was not identified in the forward genetic screens for levamisole resistance, this suggests that performing a different type of genetic screen may uncover new genes important for muscle function.

Feeding *C. elegans* bacteria that make double-stranded RNA (dsRNA) produce an RNA interference (RNAi) signal that spreads throughout the animal, causing knockdown of corresponding gene function (Timmons *et al.* 2001). Since animals treated with dsRNA do not have to be propagated and the dsRNA can be delivered post-embryonically, genome-wide RNAi screens can identify genes that are otherwise essential for viability. Prior RNAi screens that assessed approximately 10% of the genome for resistance and hypersensitivity to the acetylcholine esterase inhibitor aldicarb identified genes important for cholinergic neurotransmission and GABAergic signaling, respectively (Sieburth *et al.* 2005; Vashlishan *et al.* 2008). Here, we performed a systematic genome-wide RNAi screen for gene knockdowns that cause hypersensitivity or resistance to levamisole to identify novel regulators of postsynaptic signaling. We identified 135 gene knockdowns that resulted in altered levamisole response. Only 7% of the genes that we discovered had a previously annotated levamisole phenotype, suggesting that our screen identified new regulators of postsynaptic function.

## Materials and Methods

### Nematode culture

*C. elegans* were maintained on Nematode Growth Medium (NGM) plates with OP50 *E. coli* using standard techniques (Brenner 1974). The wild-type strain was Bristol N2. Other strains used in this study were as follows: CB1072 *unc-29(e1072)* I, ZZ17 *lev-10(x17)* I, CB407 *unc-49(e407)* III, GR1373 *eri-1(mg366)* IV, CW152 *gas-1(fc21)* X, GS2526 *arIS37* I; *mca-3(ar492) dpy-20(e1282)* IV, UDE5 *unc-63(kr98-YFP)* I; *eri-1(mg366)* IV, UDE23 *krSi2(unc-49::tagRFP)* III; *eri-1(mg366)* IV. All *C. elegans* strains were cultured at 20 °C except those with *eri-1(mg366)*, which were maintained at 15 °C.

### Levamisole time course swim assay

**Figure 1:**
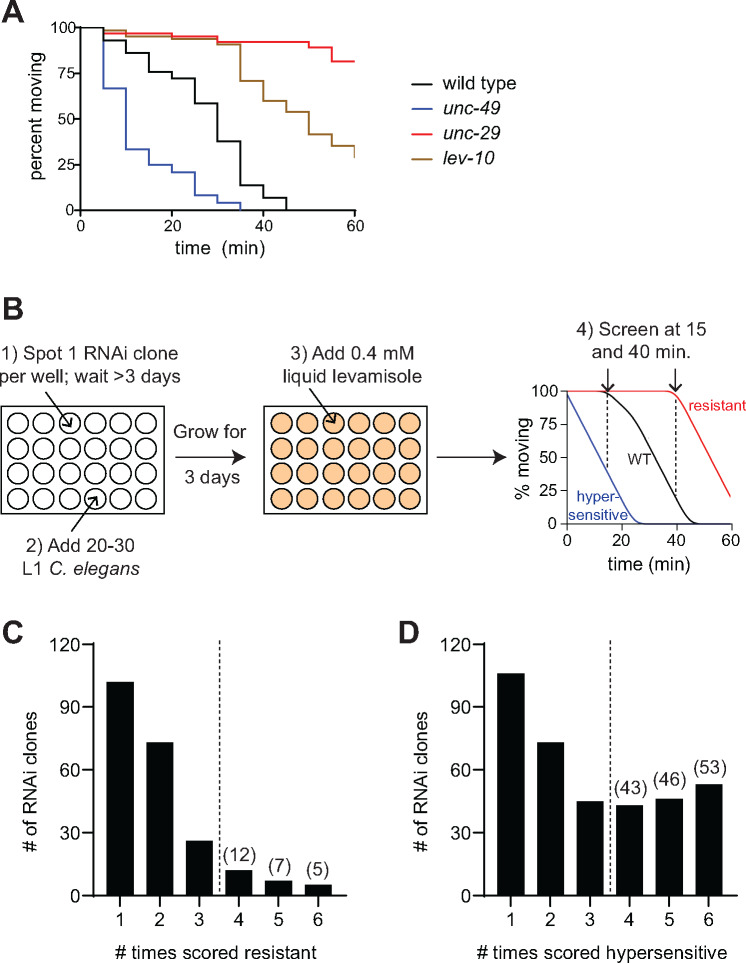
RNAi screen for altered sensitivity to levamisole (A) Time-dependent paralysis of wild-type (black), *unc-29* (red), *lev-10* (tan), and *unc-49* (blue) animals in a levamisole swim assay. Loss of *unc-29* (red) and *lev-10* (tan) caused resistance; loss of *unc-49* (blue) resulted in hypersensitivity. n≥30 per genotype in this representative experiment; p <0.0001 for all mutants compared to the wild-type control. (B) Workflow for the RNAi screen. *eri-1* L1s were fed bacteria expressing dsRNA in 24 well plates. After three days, 0.4 mM levamisole in M9 was added to each well and animals were scored for hypersensitivity after 15 minutes and resistance after 40 minutes. (C-D) After the primary screen was performed in duplicate, “hits” were screened again in quadruplicate. Gene knockdowns were considered positive if altered levamisole sensitivity was observed four or more times (p < 0.05).

**Figure 2:**
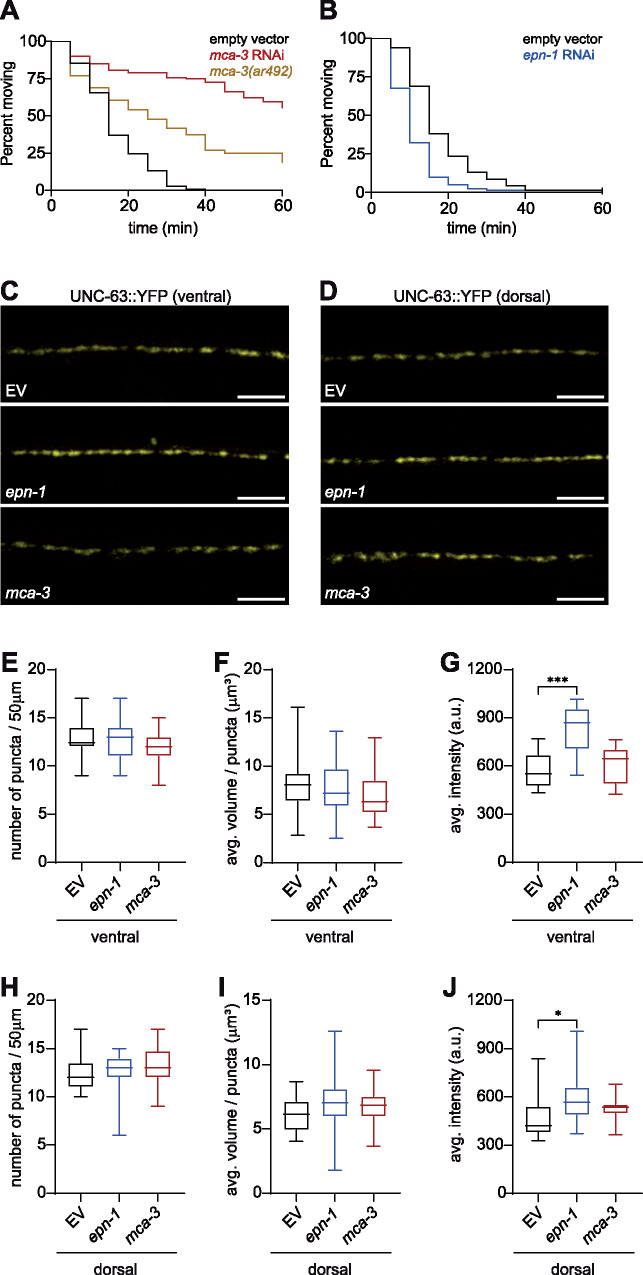
Loss of EPN-1 increases the abundance of L-AChRs (A) *mca-3* knockdown (red) and the *mca-3(ar492)* mutation (tan) both cause resistance to levamisole induced paralysis; p < 0.0001, n ≥ 50. (B) Knockdown of *epn-1* (blue) results in hypersensitivity to levamisole; p < 0.0001, n ≥ 68 (C, D) Representative images of YFP-tagged UNC-63 L-AChRs on the ventral (C) and dorsal (D) sides of animals exposed to empty vector (EV, top), *epn-1* (middle), and *mca-3* (bottom) RNAi; anterior to the left, scale bar = 10 µm. (E-J) Quantitation of the density (E, H), volume (F, I), and abundance (G, J) of UNC-63::YFP clusters at the NMJ in EV, *epn-1*, and *mca-3* knockdown animals; ventral (E-G) and dorsal (H-J) measurements are indicated. Data are shown in box plots (min to max), n ≥ 16, *** p < 0.001, one-way ANOVA with Dunnett’s post-test.

24 well-NGM plates or RNAi plates (NGM plus 1 mM IPTG and 25 µg/µL carbenicillin) with 40 µL of bacterial food source per well were prepared. In each plate, there were at least three independent wells per genotype (Figures 1A, 4F) or RNAi clone (Figures 2A, B and Figure 4 A-E); researchers who performed the assay were blinded. About twenty-first larval stage (L1) animals synchronized by bleaching were pipetted into each well. After three days, approximately 1 mL of 0.4 mM levamisole (Sigma) in M9 buffer was added to wells at intervals. The number of moving worms per well was counted every 5 minutes until all worms stopped moving or 60 minutes passed; a worm was classified as moving as long it completed at least one body bend. The total number of worms per well was recorded at the end of the assay. Data from the wells containing the same RNAi clone were pooled and used to generate Kaplan-Meier survival plots in Graph Pad Prism; log-rank (Mantel-Cox) tests were used to determine significance. At least three independent assays were conducted on different days for each genotype or RNAi clone; representative experiments are shown in the figures. For time course assays with gene knockdowns, at least three wells of empty vector control were present in every 24 well plate, as there can be variability in levamisole stock, plates animals are grown on, and scoring done by different individuals carrying out the assay.

### Levamisole sensitivity RNAi screen

The RNAi feeding screen was carried out in 24 well format as described (Kamath *et al.* 2003). Around 30 L1 *eri-1(mg366)* animals were deposited in each well. After three days of RNAi exposure at 20 °C, wells were checked for the presence of adult worms and lack of contamination. Approximately 1 mL of 0.4 mM levamisole (Sigma) in M9 buffer was added and the number of moving animals in each well was recorded at 15 and 40 minutes to identify hypersensitivity and resistance, respectively. Wells with at least 50% of the animals paralyzed at 15 minutes were marked as hypersensitive, while wells with at least 30% of the animals moving at 40 minutes were scored as resistant. Animals grown on the HT115 empty vector control were scored every day of screening. The entire genome-wide screen was performed in duplicate. Knockdowns that caused altered levamisole response twice, or once for genes with known muscle expression, were retested in quadruplicate. For the final gene list (Supplemental Table 1), the RNAi clones caused a levamisole phenotype in at least 4/6 trials, did not induce paralysis in a 60-minute swim test in water, produced a levamisole phenotype in a single time course assay, and were sequence verified.

### Fluorescence microscopy and image analysis

UDE5 *unc-63(kr98-YFP)*; *eri-1(mg366)* animals to visualize L-AChRs, and UDE23 *krSi2(unc-49::tagRFP)*; *eri-1(mg366)* animals to visualize GABA_A_ receptors (GABA_A_Rs), were grown on empty vector, *epn-1* and *mca-3* RNAi plates. Late fourth-larval stage (L4) animals were picked onto new corresponding RNAi plates, cultured for 24 hours at 20°C to produce staged adults, and immobilized with 50 mM sodium azide on 3% agar pads 10 minutes prior to imaging. Z-stack images of UNC-63::YFP receptors mid-way between the second pharyngeal bulb and the vulva were obtained with a Zeiss LSM880 confocal microscope using a 40X water objective and master gain of 870. UNC-49::tagRFP images were collected with a Zeiss LSM780 confocal microscope using a 40X water objective and master gain of 981. Z-stack images were deconvolved using Huygens Professional Software with signal-to-noise ratio set to 5 and quality threshold of 0.05. Images with no indication of movement were analyzed with Volocity software with a minimum object size of 1 µm^3^. The numbers of puncta per 50 µm segment, volume (µm^3^) of each punctum, and mean intensity (arbitrary units) of each punctum were recorded. A non-parametric one-way ANOVA with Dunnett’s multiple comparisons test (GraphPad Prism) was used to test for significance.

### ATP quantitation

ATP quantification assays were performed with the ATP Bioluminescence Assay Kit CLS II (Roche Diagnostics) as described (Palikaras and Tavernarakis 2016) with some modifications. Synchronized populations of *unc-29(e1072)* and wild-type animals, ∼1000 per 10 cm NGM plate, were exposed to the following conditions: 60 minutes no treatment, 60 minutes M9, or 60 minutes 0.4 mM levamisole in M9. Animals were washed 3x with M9 solution and 20 µL of worms were transferred to microcentrifuge tubes. 180 µL of boiling Tris-EDTA buffer (100 mM Tris, 4 mM EDTA pH 7.75) was added and samples were incubated at 100 °C for 2 minutes, sonicated for 4 minutes (Model 150 V/T Ultrasonic Homogenizer), and then centrifuged at 14,000 RPM for 10 minutes at 4 °C. 30 µL of the supernatant was added to 270 µL Tris-EDTA buffer to make 1:10 dilutions for triplicate technical replicates. ATP content was determined following the Roche ATP Bioluminescence Assay Kit CLS II protocol using a Glomax 96 Microplate Luminometer. ATP levels were normalized to total protein content (Pierce BCA protein assay kit). Six biological replicates were performed; statistical significance was determined using a non-parametric one-way ANOVA with Dunnett’s post-test.

## Results and Discussion

### An RNAi screen for altered sensitivity to levamisole

The body wall muscles receive excitatory and inhibitory inputs from cholinergic and GABAergic motor neurons, respectively. Altered time to levamisole-induced paralysis can be used to identify genes that impact the balance of postsynaptic excitatory and inhibitory signaling. While levamisole-resistant mutants have been isolated, mutations in genes required for both viability and levamisole response could not be identified in the forward genetic screens, and there has never been a genetic screen for levamisole hypersensitivity (Lewis *et al.* 1980; Rapti *et al.* 2011; Boulin *et al.* 2012; Richard *et al.* 2013; D’Alessandro *et al*. 2018). We reasoned that a genome-wide RNAi screen for gene knockdowns that cause hypersensitivity or resistance to levamisole would identify new regulators of postsynaptic function.

Traditionally, resistance or hypersensitivity to levamisole has been determined using a plate-based assay, which requires prodding animals to assess paralysis. To enable high-throughput screening, we developed a liquid levamisole swim assay performed on animals grown in 24 well plates. Compared to plate-based experiments, the liquid levamisole assay required a lower concentration of levamisole, 0.4 mM, to induce complete paralysis of wild-type animals within 45 minutes (Figure 1A). Further, the vigorous swimming of the animals in liquid allowed us to assess paralysis without physically prodding the animals. We tested mutants with known levamisole phenotypes in our new assay and found that mutations in *unc-29*, which encodes an essential L-AChR subunit and *lev-10*, which is important for L-AChR clustering, caused resistance (Figure 1A) (Lewis *et al.* 1980; Gally *et al.* 2004). The swim assay also detected hypersensitivity to levamisole in the *unc-49* GABA_A_R mutant, which has reduced inhibitory signaling (Figure 1A) (Richmond and Jorgensen 1999; Bamber *et al.* 2005). These results demonstrate that levamisole phenotypes can be observed using a swim-based levamisole assay and since this assay is less labor intensive, it is ideal for a genetic screen.

We performed liquid levamisole swim assays on animals grown on RNAi clones in 24-well plates and scored the number of animals moving at two time points to identify hypersensitivity and resistance (Figure 1B). In the primary screen, we tested animals fed clones from the *C. elegans* RNAi library generated by the Ahringer group (Kamath *et al.* 2003), representing ∼86% of the genome, in duplicate. Gene knockdowns that caused altered levamisole response twice, or once for genes with known muscle expression, were defined as “hits” and retested in quadruplicate. We identified 142 RNAi clones that caused hypersensitivity and 24 RNAi clones that produced resistance in at least four out of six trials and did not induce paralysis during a 60-minute swim test, which ensured that these animals were capable of movement for the entire assay time period (Figure 1C, D). 93% of the gene knockdowns that caused levamisole hypersensitivity in the screen paralyzed significantly faster than the control in time course experiments; 75% of the gene knockdowns that were found to elicit resistance in the screen were validated with time course assays. In total, we sequence-verified 135 of the RNAi clones that caused altered levamisole sensitivity in time course assays (Supplemental Table 1). 89% have human homologs, and of those with a defined expression pattern, 74% are muscle expressed. This indicates that we identified highly conserved genes with postsynaptic expression at the NMJ.

Although loss of genes identified in our screen produced the same levamisole phenotype when scored with the swim-based and plate-based assays (Figure 1A, Supplemental Figure 1), just 7% of the genes that we discovered were previously associated with altered levamisole sensitivity. Of the RNAi clones for nine of the genes identified in the forward genetic screen for strong levamisole resistance, we identified only a third of these, *unc-63*, *lev-10*, and *unc-22*, in our screen. While many other genes that impact levamisole sensitivity were also not identified in our screen, there are multiple possible reasons for this. First, not all of the RNAi clones in the library cause efficient knockdown (Supplemental Figure 2). Second, since RNAi exposure commenced at the first larval stage, there may have been insufficient knock down of some genes to generate an altered levamisole response by the time the animals were assayed. Third, some of the RNAi clones caused severe developmental defects that prevented us from screening the levamisole sensitivity of those animals. Fourth, we used a different levamisole assay than the forward genetic screens and thus we were unable to identify animals that initially exhibit paralysis in response to levamisole, but subsequently adapt and regain motility (Rapti *et al.* 2011; Boulin *et al.* 2012; Richard *et al.* 2013; D’Alessandro *et al*. 2018). Finally, contamination and lack of animals in a limited number of wells prevented us from screening some library clones in duplicate. Despite these limitations, our screen identified 126 new genes that exhibit a levamisole phenotype when knocked down.

Our reverse genetic screen provided unique advantages that enabled us to discover these new genes required for proper levamisole response. We were able to detect both hypersensitive and resistant phenotypes with our two-time point screen and by initiating gene knockdown after embryogenesis was complete, we were able to identify many genes otherwise essential for viability. Notably, 50% of the genes that we discovered have an annotated sterile or lethal phenotype, justifying the need for a reverse genetic screen to identify additional factors that impact levamisole response.

Here, we have performed an initial characterization of two different classes of genes identified in our screen.

### *C. elegans* Epsin regulates postsynaptic receptor abundance

During endocytosis, the plasma membrane and associated proteins are pulled toward the cytosol, membrane scission occurs, and the endocytic vesicle is internalized. Cargoes are then either targeted for degradation or recruited back to the plasma membrane via recycling endosomes to modulate synaptic strength (Park *et al.* 2004; Wang *et al.* 2008). Maintenance of the proper abundance of postsynaptic receptors is achieved by balancing the delivery of new receptors to the plasma membrane with the endocytic removal of existing receptors. Genes required for L-AChR assembly, trafficking to the cell surface, and clustering on the body wall muscles, but not internalization have been previously identified (Gally *et al.* 2004; Eimer *et al.* 2007; Gendrel *et al.* 2009; Rapti *et al.* 2011; Richard *et al.* 2013; D’Alessandro *et al*. 2018). We discovered two genes in our screen, *mca-3* and *epn-1*, which have previously described roles in endocytosis. MCA-3, a plasma membrane Ca^2+^ ATPase (PMCA), functions in clathrin-mediated endocytosis in *C. elegans* coelomocytes by recruiting endocytic machinery to the plasma membrane (Bednarek *et al.* 2007). Further, in hippocampal neurons, inhibition of PMCA2 triggers the loss of ionotropic α7-nAChRs (Gómez-Varela *et al.* 2012). Knockdown of *mca-3* caused levamisole resistance in time course assays (Figure 2A). Although null mutations in *mca-3* cause lethality, we were able to validate our results with a partial loss of function mutation and found that this generated significant levamisole resistance, though a weaker phenotype than observed with *mca-3* knockdown (Figure 2A). EPN-1 is a homolog of mammalian Epsin, which functions as an adaptor to recruit specific cargoes and induce membrane curvature that results in endocytic vesicle formation (Chen *et al.* 1998; Wendland *et al.* 1999; Ford *et al.* 2002; Wang *et al.* 2006). Knockdown of *epn-1* resulted in levamisole hypersensitivity, though we could not confirm this result with an *epn-1* mutant, as all known mutants are non-viable (Figure 2B). Both *mca-3* and *epn-1* are expressed in the body wall muscles where they could play a role in regulating the abundance of postsynaptic receptors (Bednarek *et al.* 2007; Shen *et al.* 2013).

To determine if loss of *mca-3* or *epn-1* had an impact on L-AChR abundance, we used a knock-in strain that expresses YFP-tagged UNC-63, an essential subunit of the L-AChR, from its genomic locus (Gendrel *et al.* 2009) and analyzed the distribution of UNC-63::YFP puncta in animals grown on empty vector, *mca-3*, and *epn-1* RNAi clones. We quantitated the number of puncta per 50 µm to define the quantity of postsynaptic specializations, the average volume of each puncta to determine the size of these specializations, and the average intensity to measure the relative number of receptors. Based on these parameters, loss of *mca-3* did not affect L-AChR abundance or localization (Figure 2C-J). Therefore, the levamisole-resistant phenotype associated with loss of *mca-3* cannot be explained by a reduced number of L-AChRs. However, loss of *epn-1* caused a significant increase in UNC-63::YFP intensity, without altering puncta number or size, on both the dorsal and ventral sides of the animals (Figure 2C-J). This increase in the number of L-AChRs at or near the cell surface is consistent with the levamisole-hypersensitive phenotype observed in *epn-1* knockdown animals and could be the result of either different L-AChR protein levels or altered insertion into the membrane. Our results suggest that EPN-1 may play a role in the endocytosis of L-AChRs from the plasma membrane without affecting the number or size of postsynaptic specializations.

**Figure 3:**
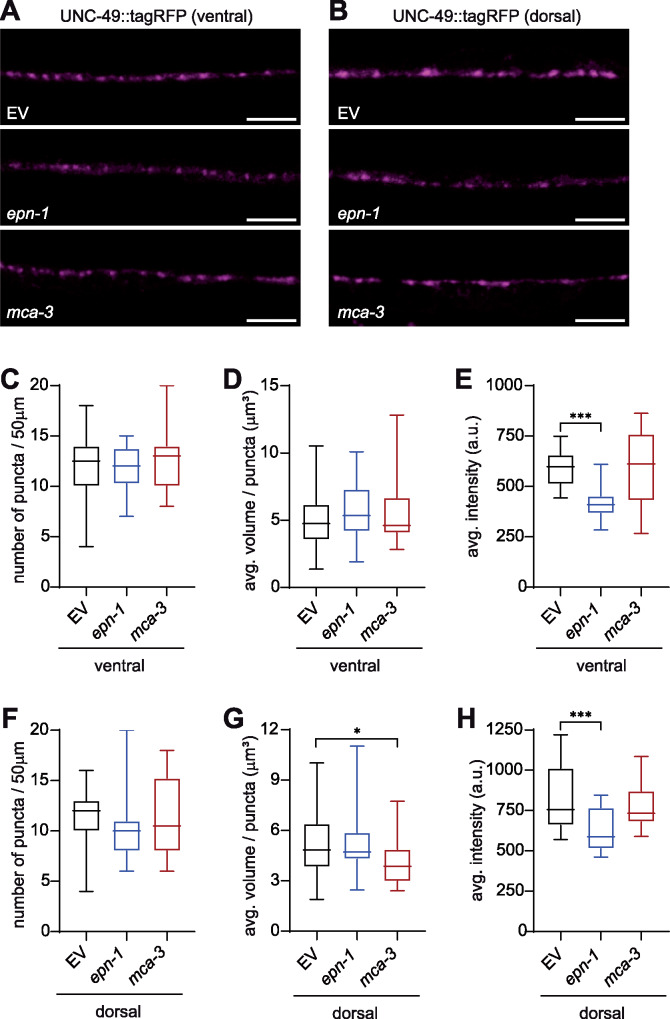
Loss of EPN-1 decreases postsynaptic GABA_A_R abundance (A, B) Representative images of RFP-tagged UNC-49 GABA_A_Rs on the ventral (A) and dorsal (B) sides of animals exposed to empty vector (EV, top), *epn-1* (middle), and *mca-3* (bottom) RNAi; anterior to the left, scale bar = 10 µm. (C-H) Quantitation of UNC-49::tagRFP density (C, F), volume (D, G), and abundance (E, H) on the body wall muscles of EV, *epn-1*, and *mca-3* knockdown animals; ventral (C-E) and dorsal (F-H) measurements are indicated. Data are shown in box plots (min to max), n ≥ 17, * p < 0.05, *** p < 0.001, one-way ANOVA with Dunnett’s post-test.

Defects in GABA signaling can disrupt the excitatory-inhibitory balance in the body wall muscles, resulting in altered levamisole response (Vashlishan *et al.* 2008; Krajacic *et al.* 2013; Kowalski *et al.* 2014). We reasoned that an increase in postsynaptic UNC-49 GABA_A_Rs and thus inhibitory signaling could lead to a levamisole-resistant phenotype such as that observed with loss of *mca-3*. In fact, *cup-5*, a mutant identified along with *mca-3* (*cup-7*) in a forward genetic screen for coelomocyte uptake defective mutants regulates plasma membrane levels of GABA_A_Rs (Fares and Greenwald 2001; Davis *et al.* 2010). Using a transgenic strain that expresses RFP-tagged UNC-49 at single copy (Pinan-Lucarré *et al.* 2014), we analyzed the distribution of postsynaptic UNC-49::tagRFP receptor puncta in animals grown on empty vector, *mca-3*, and *epn-1* RNAi clones. We found that, for the most part, loss of *mca-3* did not alter GABA_A_R abundance or localization, though a small, yet significant decrease in the average volume of GABA_A_R puncta was observed on the dorsal side (Figure 3A-H). Surprisingly, analysis of GABA_A_Rs in the *epn-1* knockdown showed a significant decrease in UNC-49::tagRFP intensity compared to the control, with no effect on the number or size of the puncta (Figure 3A-H). These results suggest that EPN-1 regulates the abundance of both L-AChRs and GABA_A_Rs at the NMJ.

Both the increase in L-AChRs and decrease in GABA_A_Rs on the body wall muscles likely contribute to the levamisole-hypersensitive phenotype observed in *epn-1* knockdown animals. However, the mechanism by which EPN-1 has opposing effects on the abundance of two different types of postsynaptic receptors, impacting both excitatory and inhibitory synapses, is not known. EPN-1 has been shown to be expressed in multiple cell types including body wall muscles and neurons (Shen *et al.* 2013). As loss of *epn-1* increases L-AChR abundance, EPN-1 may act directly in the body wall muscles to regulate the endocytosis of these receptors. How EPN-1 controls the levels of GABA_A_Rs is likely less straightforward. Mutants with GABA transmission defects can display levamisole hypersensitivity (Vashlishan *et al.* 2008; Kowalski *et al.* 2014). Further, the *C. elegans* Punctin MADD-4B, secreted by GABAergic neurons, is required for appropriate recruitment of GABA_A_Rs to the NMJ (Pinan-Lucarré *et al.* 2014; Tu *et al.* 2015). Thus, it is possible that EPN-1 plays an endocytic role in GABAergic neurons, leading to a compensatory effect on postsynaptic GABA_A_Rs. Future studies will define the EPN-1 site of action, subcellular distribution, and effect on other synaptic markers as well as whether postsynaptic receptor abundance is regulated by clathrin-mediated or clathrin-independent endocytosis.

### Disruption of cellular ATP levels alters levamisole sensitivity

**Figure 4:**
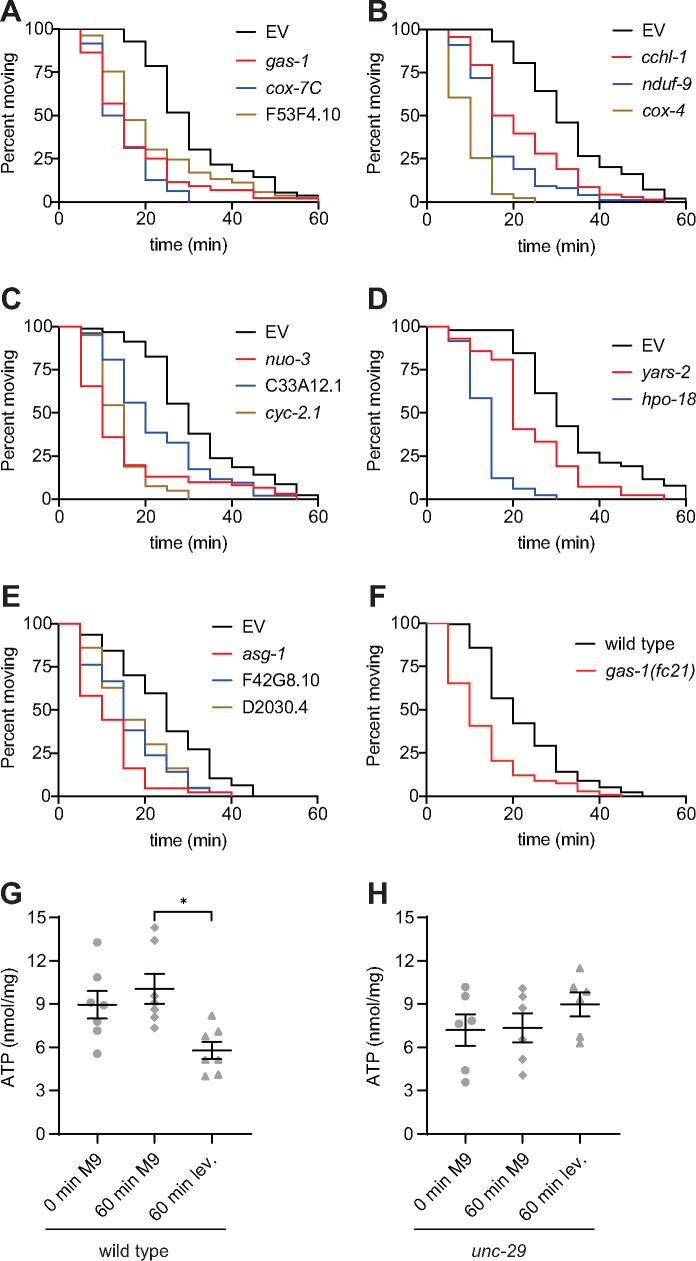
Levamisole exposure reduces cellular ATP (A-E) A functional class with genes required for ATP synthesis was identified in the RNAi screen for altered sensitivity to levamisole. Knockdown of *gas-1* (red), *cox-7c* (blue), and F53F4.10 (tan) in (A), c*chl-1* (red), *nduf-9* (blue), and *cox-4* (tan) in (B), *nuo-3* (red), C33A12.1 (blue), and *cyc-2.1* (tan) in (C), *yars-2* (red), and *hpo-18* (blue) in (D), and *asg-1* (red), F42G8.10 (blue), and D2030.4 (tan) in (E) causes hypersensitivity to levamisole compared to empty vector (EV, black) in time course assays; p < 0.001 for all knockdowns. (F) Like the *gas-1* knockdown in (A), *gas-1(fc21)* (red) exhibits hypersensitivity to levamisole-induced paralysis compared to wild-type (black); p < 0.001. (G, H) 60 minutes of exposure to 0.4 mM levamisole (lev) causes a significant decrease in ATP (* p < 0.05, one-way ANOVA with Dunnett’s post-test) in wild-type (G), but not *unc-29* L-AChR mutants (H); data are the mean ± SE, each individual measurement is shown.

Although levamisole has been used for decades to identify mutants with defects in postsynaptic signaling and immobilize animals for microscopy, the mechanism by which treatment with this pharmacological agent results in paralysis and death has not been fully defined. L-AChR activation leads to opening of the voltage-gated Ca^2+^ channel EGL-19, muscle depolarization, and, ultimately, Ca^2+^ release from intracellular stores through the ryanodine receptor UNC-68 (Liu *et al.* 2011). Ca^2+^ binds to troponin, causing a shift in the position of tropomyosin and increasing the probability that myosin will bind the thin filament to produce force to generate muscle contraction. The sarco-endoplasmic reticulum Ca^2+^ ATPase (SERCA) works in concert with plasma membrane Ca^2+^ ATPases (PMCAs) and Na^+^/Ca^2+^ exchangers to lower cytoplasmic Ca^2+^ levels, which terminates contraction (Clapham 2007; Martin and Richmond 2018). If there is a decline in cellular ATP levels, this prevents Ca^2+^ from being pumped out of the cytoplasm, causing the muscles to remain contracted until Ca^2+^-dependent proteases induce muscle degeneration and finally relaxation in death (Galimov *et al.* 2018). It has been hypothesized that levamisole-induced paralysis and death could be the result of cellular metabolic catastrophe, a condition in which ATP depletion prevents muscle function (Lewis *et al.* 1980). We discovered that knockdown of 14 genes predicted to play a role in ATP synthesis, *gas-1*, *cox-7c*, c*chl-1*, *nduf-9*, *cox-4*, *nuo-3*, C33A12.1, *cyc-2.1*, *yars-2*, *hpo-18*, *asg-1*, F53F4.10, F42G8.10, and D2030.4, caused levamisole hypersensitivity both in our genetic screen and time course assays (Figure 4A-E). While most of these genes are essential for viability (Supplemental Table 1), we were able to validate the levamisole-hypersensitivity phenotype with a *gas-1* loss of function mutant, which has defects in Complex I of the mitochondrial electron transport chain (Figure 4F) (Kayser *et al.* 2001). These results suggest that reduced ATP synthesis increases sensitivity to levamisole, accelerating death.

To determine if levamisole exposure leads to metabolic exhaustion, we quantitated ATP levels in animals that were washed off plates, swimming in M9 buffer for 60 minutes, or swimming in 0.4 mM levamisole for 60 minutes. While swimming in buffer had no effect on measured ATP, addition of levamisole significantly decreased ATP levels (Figure 4G). This was due to signaling through L-AChRs since levamisole did not have an impact on ATP levels in the *unc-29* L-AChR mutant (Figure 4H). This shows that levamisole exposure decreases ATP in cells that express L-AChRs and provides evidence for levamisole-induced metabolic catastrophe.

It is possible that knockdown of some seemingly unrelated genes identified in our screen may result in hypersensitivity to levamisole due to reduced cellular ATP and future studies will test this hypothesis. Loss of *pat-4* (Integrin Linked Kinase), *tln-1* (Talin I), *unc-73* (Trio), and *unc-60* (Cofilin) causes fragmentation of the muscle mitochondrial network, while loss of *prmt-1* (Protein Arginine Methyltransferase I) has been shown to reduce ATP synthesis (Etheridge *et al.* 2015; Sha *et al.* 2017). Further, we reason that mutants with reduced ATP usage would produce a resistant phenotype. SERCA and PMCA together utilize >30% of muscle produced ATP to pump Ca^2+^ out of the cytoplasm (Homsher 1987). Since release of Ca^2+^ from intracellular stores through ryanodine receptors dictates the amount of ATP used by Ca^2+^ ATPases, ATP usage can be attenuated by decreasing Ca^2+^ release from intracellular stores. This provides a possible explanation for the levamisole-resistant phenotype observed in the *unc-68* ryanodine receptor mutant (Lewis *et al.* 1980). SERCAs translocate two Ca^2+^ ions into the sarco-endoplasmic reticulum lumen for each molecule of ATP hydrolyzed, while PMCAs translocate a single Ca^2+^ ion per ATP (Inesi and De Meis 1989; Hao *et al.* 1994). Thus, loss of the PMCA MCA-3 may reduce ATP usage, enabling the more efficient SERCA SCA-1 to continue to pump Ca^2+^ into the sarco-endoplasmic reticulum and leading to the levamisole resistance observed in the *mca-3* mutant. Consequently, loss of genes that affect either ATP synthesis or usage could impact levamisole sensitivity.

## Conclusion

Here, we conducted the first genome-wide reverse genetic screen for postsynaptic regulators. In summary, we identified 135 gene knockdowns that caused altered sensitivity to the L-AChR agonist levamisole, describing a levamisole phenotype for 93% of these genes for the first time. We discovered novel genes because this was the first genetic screen capable of detecting levamisole hypersensitivity and genes that impact both levamisole response and viability. One gene that caused levamisole hypersensitivity when knocked down and lethality when knocked out is *epn-1*. Our work shows that loss of *epn-1* impacts postsynaptic function by having opposing effects on the abundance of postsynaptic L-AChRs and GABA_A_Rs. Notably, our screen also identified *C. elegans* homologs of genes mutated in congenital myasthenic syndrome (AChR), congenital muscular dystrophy (Mannose-1-phosphate guanyltransferase beta), congenital myopathy (Cofilin), myotonic dystrophy (ELAV-type RNA binding protein), and mitochondrial myopathy (NADH dehydrogenase) (Lu *et al.* 1999; Milne and Hodgkin 1999; Agrawal *et al.* 2007; Ockeloen *et al.* 2012; Carss *et al.* 2013; Engel *et al.* 2015; Schubert and Vilarinho 2020). This suggests that altered response to levamisole can be used broadly to discover and study genes that impact muscle function.

A functional class of genes known to impact ATP synthesis was also identified in our screen. This led us to discover that levamisole treatment decreased cellular ATP, which likely leads to metabolic exhaustion. Levamisole is commonly used by members of the *C. elegans* community to immobilize animals for imaging experiments. Thus, our results have broad significance, as understanding the consequences of levamisole exposure is important for researchers who use this as a chemical anesthetizing agent.

## References

[jkab047-B1] Achanzar WE , Ward S. 1997. A nematode gene required for sperm vesicle fusion. J. Cell Sci. 110:1073–1081. 9175703 10.1242/jcs.110.9.1073

[jkab047-B2] Agrawal PB , Greenleaf RS, Tomczak KK, Lehtokari VL, Wallgren-Pettersson C, et al 2007. Nemaline myopathy with minicores caused by mutation of the CFL2 gene encoding the skeletal muscle actin-binding protein, cofilin-2. Am. J. Hum. Genet. 80:162–167. 10.1086/510402 17160903 PMC1785312

[jkab047-B3] Bamber BA , Richmond JE, Otto JF, Jorgensen EM. 2005. The composition of the GABA receptor at the Caenorhabditis elegans neuromuscular junction. Br. J. Pharmacol. 144:502–509. 10.1038/sj.bjp.0706052 15655525 PMC1576029

[jkab047-B4] Bednarek EM , Schaheen L, Gaubatz J, Jorgensen EM, Fares H. 2007. The plasma membrane calcium ATPase MCA-3 is required for clathrin-mediated endocytosis in scavenger cells of Caenorhabditis elegans. Traffic. 8:543–553. 10.1111/j.1600-0854.2007.00547.x 17343680

[jkab047-B5] Boulin T , Rapti G, Briseño-Roa L, Stigloher C, Richmond JE, et al 2012. Positive modulation of a Cys-loop acetylcholine receptor by an auxiliary transmembrane subunit. Nat Neurosci. 15:1374–1381. 10.1038/nn.3197 22922783

[jkab047-B6] Brenner S. 1974. The genetics of Caenorhabditis elegans. Genetics. 77:71–94. 4366476 10.1093/genetics/77.1.71 PMC1213120

[jkab047-B7] Carss KJ , Stevens E, Foley AR, Cirak S, Riemersma M, et al 2013. Mutations in GDP-mannose pyrophosphorylase b cause congenital and limb-girdle muscular dystrophies associated with hypoglycosylation of α-dystroglycan. Am. J. Hum. Genet. 93:29–41. 10.1016/j.ajhg.2013.05.009 23768512 PMC3710768

[jkab047-B8] Chen H , Fre S, Slepnev VI, Capua MR, Takei K, et al 1998. Epsin is an EH-domain-binding protein implicated in clathrin-mediated endocytosis. Nature. 394:793–797. 10.1038/29555 9723620

[jkab047-B9] Clapham DE. 2007. Calcium Signaling. Cell. 131:1047–1058. 10.1016/j.cell.2007.11.028 18083096

[jkab047-B10] D'Alessandro M , Richard M, Stigloher C, Gache V, Boulin T, et al 2018. CRELD1 is an evolutionarily-conserved maturational enhancer of ionotropic acetylcholine receptors. Elife. 7:e39649. 10.7554/eLife.39649 30407909 PMC6245729

[jkab047-B11] Davis KM , Sturt BL, Friedmann AJ, Richmond JE, Bessereau JL, et al 2010. Regulated lysosomal trafficking as a mechanism for regulating GABAA receptor abundance at synapses in Caenorhabditis elegans. Mol. Cell. Neurosci. 44:307–317. 10.1016/j.mcn.2010.04.002 20403442

[jkab047-B12] Eimer S , Gottschalk A, Hengartner M, Horvitz HR, Richmond J, et al 2007. Regulation of nicotinic receptor trafficking by the transmembrane Golgi protein UNC-50. Embo J. 26:4313–4323. 10.1038/sj.emboj.7601858 17853888 PMC2034668

[jkab047-B13] Engel AG , Shen X-M, Selcen D, Sine SM. 2015. Congenital myasthenic syndromes: pathogenesis, diagnosis, and treatment. Lancet Neurol. 14:420–434. 10.1016/S1474-4422(14)70201-7 25792100 PMC4520251

[jkab047-B14] Etheridge T , Rahman M, Gaffney CJ, Shaw D, Shephard F, et al 2015. The integrin-adhesome is required to maintain muscle structure, mitochondrial ATP production, and movement forces in Caenorhabditis elegans. FASEB J. 29:1235–1246. 10.1096/fj.14-259119 25491313 PMC4396603

[jkab047-B15] Fares H , Greenwald I. 2001. Genetic analysis of endocytosis in caenorhabditis elegans: Coelomocyte uptake defective mutants. Genetics. 159:133–145. 11560892 10.1093/genetics/159.1.133 PMC1461804

[jkab047-B16] Ford MGJ , Mills IG, Peter BJ, Vallis Y, Praefcke GJK, et al 2002. Curvature of clathrin-coated pits driven by epsin. Nature. 419:361–366. 10.1038/nature01020 12353027

[jkab047-B17] Galimov ER , Pryor RE, Poole SE, Benedetto A, Pincus Z, et al 2018. Coupling of rigor mortis and intestinal necrosis during C. elegans organismal death. Cell Rep. 22:2730–2741. 10.1016/j.celrep.2018.02.050 29514100 PMC5863043

[jkab047-B18] Gally C , Eimer S, Richmond JE, Bessereau JL. 2004. A transmembrane protein required for acetylcholine receptor clustering in Caenorhabditis elegans. Nature. 431:578–582. 10.1038/nature02893 15457263 PMC3781939

[jkab047-B19] Gendrel M , Rapti G, Richmond JE, Bessereau JL. 2009. A secreted complement-control-related protein ensures acetylcholine receptor clustering. Nature. 461:992–996. 10.1038/nature08430 19794415

[jkab047-B20] Gieseler K. 2017. Development, structure, and maintenance of C. elegans body wall muscle. WormBook. 1–59. 10.1895/wormbook.1.81.2 PMC5410635 27555356

[jkab047-B21] Gómez-Varela D , Schmidt M, Schoellerman J, Peters EC, Berg DK. 2012. PMCA2 via PSD-95 controls calcium signaling by α7-containing nicotinic acetylcholine receptors on aspiny interneurons. J. Neurosci. 32:6894–6905. 10.1523/JNEUROSCI.5972-11.2012 22593058 PMC3369694

[jkab047-B22] Hao L , Rigaud JL, Inesi G. 1994. Ca2+/H+ countertransport and electrogenicity in proteoliposomes containing erythrocyte plasma membrane Ca-ATPase and exogenous lipids. J. Biol. Chem. 269:14268–14275. 8188711

[jkab047-B23] Homsher E. 1987. Muscle enthalpy production and its relationship to actomyosin ATPase. Annu. Rev. Physiol. 49:673–690. 10.1146/annurev.physiol.49.1.673 2952055

[jkab047-B24] Inesi G , De Meis L. 1989. Regulation of steady state filling in sarcoplasmic reticulum. Roles of back-inhibition, leakage, and slippage of the calcium pump. J. Biol. Chem. 264:5929–5936. 2522442

[jkab047-B25] Kamath RS , Fraser AG, Dong Y, Poulin G, Durbin R, et al 2003. Systematic functional analysis of the Caenorhabditis elegans genome using RNAi. Nature. 421:231–237. 10.1038/nature01278 12529635

[jkab047-B26] Kayser EB , Morgan PG, Hoppel CL, Sedensky MM. 2001. Mitochondrial expression and function of GAS-1 in Caenorhabditis elegans. J. Biol. Chem. 276:20551–20558. 10.1074/jbc.M011066200 11278828

[jkab047-B27] Kowalski JR , Dube H, Touroutine D, Rush KM, Goodwin PR, et al 2014. The anaphase-promoting complex (APC) ubiquitin ligase regulates GABA transmission at the C. elegans neuromuscular junction. Mol. Cell. Neurosci. 58:62–75. 10.1016/j.mcn.2013.12.001 24321454 PMC4036811

[jkab047-B28] Krajacic P , Pistilli EE, Tanis JE, Khurana TS, Lamitina ST. 2013. FER-1/Dysferlin promotes cholinergic signaling at the neuromuscular junction in C. elegans and mice. Biol. Open. 2:1245–1252. 10.1242/bio.20135637 24244862 PMC3828772

[jkab047-B29] Lewis JA , Wu CH, Berg H, Levine JH. 1980. The genetics of levamisole resistance in the nematode Caenorhabditis elegans. Genetics. 95:905–928. 7203008 10.1093/genetics/95.4.905 PMC1214276

[jkab047-B30] Liu P , Ge Q, Chen B, Salkoff L, Kotlikoff MI, et al 2011. Genetic dissection of ion currents underlying all-or-none action potentials in C. elegans body-wall muscle cells. J. Physiol. 589:101–117. 21059759 10.1113/jphysiol.2010.200683 PMC3039263

[jkab047-B31] Lu X , Timchenko NA, Timchenko LT. 1999. Cardiac elav-type RNA-binding protein (ETR-3) binds to RNA CUG repeats expanded in myotonic dystrophy. Hum. Mol. Genet. 8:53–60. 10.1093/hmg/8.1.53 9887331

[jkab047-B32] Mahjneh I , Lochmüller H, Muntoni F, Abicht A. 2013. DOK7 limb-girdle myasthenic syndrome mimicking congenital muscular dystrophy. Neuromuscul. Disord. 23:36–42. 10.1016/j.nmd.2012.06.355 22884442

[jkab047-B33] Martin AA , Richmond JE. 2018. The sarco(endo)plasmic reticulum calcium ATPase SCA-1 regulates the Caenorhabditis elegans nicotinic acetylcholine receptor ACR-16. Cell Calcium. 72:104–115. 10.1016/j.ceca.2018.02.005 29748129

[jkab047-B34] Milne CA , Hodgkin J. 1999. ETR-1, a homologue of a protein linked to myotonic dystrophy, is essential for muscle development in Caenorhabditis elegans. Curr. Biol. 9:1243–1246. 10.1016/s0960-9822(99)80504-1 10556089

[jkab047-B35] Montagnese F , Klupp E, Karampinos DC, Biskup S, Gläser D, et al 2017. Two patients with GMPPB mutation: The overlapping phenotypes of limb-girdle myasthenic syndrome and limb-girdle muscular dystrophy dystroglycanopathy. Muscle Nerve. 56:334–340. 10.1002/mus.25485 27874200

[jkab047-B36] Ockeloen CW , Gilhuis HJ, Pfundt R, Kamsteeg EJ, Agrawal PB, et al 2012. Congenital myopathy caused by a novel missense mutation in the CFL2 gene. Neuromuscul. Disord. 22:632–639. 10.1016/j.nmd.2012.03.008 22560515 PMC3377783

[jkab047-B37] Palikaras K , Tavernarakis N. 2016. Intracellular assessment of ATP levels in Caenorhabditis elegans. BIO-PROTOCOL. 6:e22048. 10.21769/bioprotoc.2048 28194429 PMC5303341

[jkab047-B38] Park M , Penick EC, Edwards JG, Kauer JA, Ehlers MD. 2004. Recycling endosomes supply AMPA receptors for LTP. Science. 305:1972–1975. 10.1126/science.1102026 15448273

[jkab047-B39] Pinan-Lucarré B , Tu H, Pierron M, Cruceyra PI, Zhan H, et al 2014. C. elegans punctin specifies cholinergic versus GABAergic identity of postsynaptic domains. Nature. 511:466–470. 10.1038/nature13313 24896188

[jkab047-B40] Rapti G , Richmond J, Bessereau JL. 2011. A single immunoglobulin-domain protein required for clustering acetylcholine receptors in C. elegans. Embo J. 30:706–718. 10.1038/emboj.2010.355 21252855 PMC3041951

[jkab047-B41] Richard M , Boulin T, Robert VJP, Richmond JE, Bessereau JL. 2013. Biosynthesis of ionotropic acetylcholine receptors requires the evolutionarily conserved ER membrane complex. Proc Natl Acad Sci Usa. 110:E1055–63. 10.1073/pnas.1216154110 23431131 PMC3600456

[jkab047-B42] Richmond JE , Jorgensen EM. 1999. One GABA and two acetylcholine receptors function at the C. elegans neuromuscular junction. Nat Neurosci. 2:791–798. 10.1038/12160 10461217 PMC2585773

[jkab047-B43] Rodríguez Cruz PM , Sewry C, Beeson D, Jayawant S, Squier W, et al 2014. Congenital myopathies with secondary neuromuscular transmission defects; a case report and review of the literature. Neuromuscul. Disord. 24:1103–1110. 10.1016/j.nmd.2014.07.005 25127990

[jkab047-B44] Schubert MB , Vilarinho L. 2020. Molecular basis of Leigh syndrome: a current look. Orphanet J. Rare Dis. 15:31. 10.1186/s13023-020-1297-9 PMC6990539 31996241

[jkab047-B45] Sha L , Daitoku H, Araoi S, Kaneko Y, Takahashi Y, et al 2017. Asymmetric arginine dimethylation modulates mitochondrial energy metabolism and homeostasis in Caenorhabditis elegans. Mol. Cell. Biol. 37:e00504. 10.1128/mcb.00504-16 27994012 PMC5335503

[jkab047-B46] Shen Q , He B, Lu N, Conradt B, Grant BD, et al 2013. Phagocytic receptor signaling regulates clathrin and epsinmediated cytoskeletal remodeling during apoptotic cell engulfment in C. elegans. Development. 140:3230–3243. 10.1242/dev.093732 23861060 PMC3931732

[jkab047-B47] Sieburth D , Ch'ng QLim, Dybbs M, Tavazoie M, Kennedy S, et al 2005. Systematic analysis of genes required for synapse structure and function. Nature. 436:510–516. 10.1038/nature03809 16049479

[jkab047-B48] Timmons L , Court DL, Fire A. 2001. Ingestion of bacterially expressed dsRNAs can produce specific and potent genetic interference in Caenorhabditis elegans. Gene. 263:103–112. 10.1016/S0378-1119(00)00579-5 11223248

[jkab047-B49] Touroutine D , Fox RM, Von Stetina SE, Burdina A, Miller DM, et al 2005. *acr-16* encodes an essential subunit of the levamisole-resistant nicotinic receptor at the Caenorhabditis elegans neuromuscular junction. J. Biol. Chem. 280:27013–27021. 10.1074/jbc.M502818200 15917232

[jkab047-B50] Tu H , Pinan-Lucarré B, Ji T, Jospin M, Bessereau JL. 2015. C. elegans punctin clusters GABAA receptors via neuroligin binding and UNC-40/DCC recruitment. Neuron. 86:1407–1419. 10.1016/j.neuron.2015.05.013 26028575

[jkab047-B51] Vashlishan AB , Madison JM, Dybbs M, Bai J, Sieburth D, et al 2008. An RNAi screen identifies genes that regulate GABA synapses. Neuron. 58:346–361. 10.1016/j.neuron.2008.02.019 18466746

[jkab047-B52] Wang H , Traub LM, Weixel KM, Hawryluk MJ, Shah N, et al 2006. Clathrin-mediated endocytosis of ENaC: Role of epsin. J Biol Chem. 281:14129–14135. 10.1074/jbc.M512511200 16574660

[jkab047-B53] Wang Z , Edwards JG, Riley N, Provance DW, Karcher R, et al 2008. Myosin Vb mobilizes recycling endosomes and AMPA receptors for postsynaptic plasticity. Cell. 135:535–548. 10.1016/j.cell.2008.09.057 18984164 PMC2585749

[jkab047-B54] Wendland B , Steece KE, Emr SD. 1999. Yeast epsins contain an essential N-terminal ENTH domain, bind clathrin and are required for endocytosis. Embo J. 18:4383–4393. 10.1093/emboj/18.16.4383 10449404 PMC1171513

